# Study on the spatiotemporal distribution of Neolithic settlement clusters based on complex social evolution: Evidence from the Dongting Lake Basin

**DOI:** 10.1371/journal.pone.0358977

**Published:** 2026-10-13

**Authors:** Xinyu Du, Jun Cai, Wei Gong, Yuxi Liu, Chenmingyang Jiang, Huirong Hu, Shiliang Liu, Qibing Chen

**Affiliations:** 1 College of Landscape Architecture, Sichuan Agricultural University, Chengdu, China; 2 Hunan Institute of Nuclear Agricultural Science and Space Mutation Breeding, Hunan Academy of Agricultural Sciences, Changsha, China; 3 Xiangtan City Urban Management and Comprehensive Law Enforcement Bureau, Landscape Architecture Center, Xiangtan, China; Maria Curie-Sklodowska University: Uniwersytet Marii Curie-Sklodowskiej, POLAND

## Abstract

Settlement aggregation has been a fundamental feature of human sedentary societies and provides important evidence for understanding changes in prehistoric social organization. However, previous studies of prehistoric settlement clusters have focused predominantly on spatial division and morphological structure, while comparatively little attention has been paid to their long-term spatiotemporal evolution at the watershed scale. This study investigates the spatiotemporal evolution of Neolithic settlement clusters in the Dongting Lake Basin and its implications for the development of social complexity. A total of 1,101 Neolithic settlement sites analyzed using Density-Based Spatial Clustering of Applications with Noise (DBSCAN) algorithm, Arc Geographic Information Systems (ArcGIS), and a Spatiotemporal Object Model. The findings reveal that settlement clusters were initially concentrated primarily in the northwestern part of the Dongting Lake basin and subsequently expanded toward the areas surrounding Dongting Lake. Their spatial organization of settlement clusters gradually evolved from a pattern characterized by “small settlements and loose clustering” into a multi-level hierarchical structure consisting of “large central settlements–medium-sized secondary settlements–small local settlements.” The spatial center of the settlement clusters shifted southward at a relatively rapid pace, whereas its shifts toward the northwestern part and peripheral areas of Dongting Lake occurred more slowly. producing an overall V-shaped trajectory. The warm and humid climatic conditions, together with proximity to rivers and lakeside plains, provided favorable environmental settings for settlement development. Over time, settlement sizes generally increased, and the clusters underwent migration and differentiation, indicating increased social complexity. These findings provide new insights into the long-term spatiotemporal organization of Neolithic settlement systems in the middle Yangtze River region and contribute to a better understanding of interactions among environmental change, settlement dynamics, and the development of prehistoric social complexity.

## Introduction

Long-term human aggregation and sedentary living contributed to the emergence and development of individual settlements. As settlements increased in number and became spatially concentrated within particular geographical area, settlement clusters gradually emerged [1]. Rather than representing simple aggregations of individual settlements, settlement clusters reflects the spatial organization, interactions, and functional relationships among settlements within a defined geographical area. Their formation and development are shaped by the interplay of environmental conditions, resource availability, and sociocultural interactions [2]. Investigating the spatiotemporal evolution of settlement clusters can therefore provide important insights into changing human- environment interactions, settlement organization, and the development of social complexity during the Neolithic age.

Since the late 19th century, archaeologists have increasingly focused on settlement archaeology. Nevertheless, research has largely concentrated on the excavation of individual settlements and the establishment of chronological sequences, without yet formulating a systematic concept of “settlement clusters.” [3] Beginning in the 1940s, Gordon R. Willey, a pioneering settlement archaeologist, carried out systematic surface surveys of hundreds of settlement sites dating to various periods in Peru’s Huilluco Valley. He examined their spatial distribution, variations in scale, and relationships with the natural environment in conjunction with broader socio-political developments. This work laid the foundation for key components of settlement cluster analysis, including spatial distribution, hierarchical organization, functional differentiation, and historical evolution [4]. Early approaches to the study of settlement clusters were largely based on the analysis of excavated remains and artifacts within a given region, using geographical similarities to identify and delineate groups of settlements. With the introduction of ArcGIS into settlement archaeology, however, the precision and visualization of spatial clustering have been significantly improved. Furthermore, ArcGIS facilitates deeper investigation into the dynamic interactions involved in the spatial evolution of settlement clusters, as well as their relationship with the surrounding environment [5–7]. Since the beginning of the 21st century, the integrated application of mathematical models from statistics and ArcGIS has progressively emerged as a prominent focus in settlement archaeology research. Methods such as the K-means clustering algorithm [8], kernel density [9], Voronoi [10], and DBSCAN have been introduced to delineate spatial boundaries of settlement clusters [11]. In light of the inherently uneven spatial distribution characteristic of settlement sites, this study adopts the DBSCAN method for the spatial segmentation of Neolithic settlement clusters.

In terms of research content, previous studies on Neolithic settlement clusters have mainly concentrated on morphological structures [12] and social organization patterns [13], or on the formation of peripheral small settlement clusters emanating from individual large settlements. By contrast, research exploring the spatial distribution patterns and evolutionary processes of settlement clusters at the watershed scale remains relatively underdeveloped. Furthermore, previous research has predominantly concentrated on the spatial distribution of settlement clusters within individual cultural phases, whereas investigations into the spatiotemporal evolution of such clusters across successive cultural phases and their underlying influencing factors remain relatively limited. From a regional perspective, research on settlement clusters has predominantly centered on the Yellow River basin [14], the Central Plains [15], the Hai-Dai region [16], the area surrounding Songshan [17], and the middle reaches of the Yangtze River [18]. In contrast, studies focusing on the Dongting Lake Basin—an area characterized by a rich sequence of Neolithic cultural phases—remain relatively limited. Existing research in this region has largely addressed two main aspects: prehistoric settlement patterns and sociocultural processes [19]. To date, no study has systematically investigated the spatiotemporal evolution of settlement clusters within the Dongting Lake Basin or examined the underlying factors driving this evolution. Therefore, this study will address a research gap by examining the spatiotemporal evolution of settlement clusters. It seeks to elucidate the spatial distribution patterns, social evolutionary trajectories, and natural environmental characteristics of Neolithic settlement clusters in the Dongting Lake Basin, thereby offering new analytical perspectives and theoretical frameworks for research on prehistoric settlements in this region.

## Materials and methods

### Study area

The Dongting Lake Basin is located south of the Jing River in the middle reaches of the Yangtze River, between latitudes 24°40’-30°05’ north and longitudes 107°10’-114°15’ east [20]. The topography is tilted from southwest and south to the central and northeastern part of the country, generally in the shape of a horseshoe [21]. The entire basin consists of the Dongting Lake area and four major sub-basins of the Xiangjiang River, Zishui River, Yuan River and Li River (Fig 1). The Dongting Lake Basin spans a total area of 263,000 square kilometers, encompassing regions within Hunan Province, Hubei Province, Chongqing Municipality, Guizhou Province, Guangxi Zhuang Autonomous Region, Jiangxi Province, and Guangdong Province. From the perspective of the spatial distribution of settlement sites across provinces, the counts are as follows: Hunan Province has 997 settlement sites, Hubei Province has 64, Jiangxi Province has 3, Guangxi Zhuang Autonomous Region has 12, Chongqing Municipality has 3, Guizhou Province has 22, and Guangdong Province has 0. Among these, Hunan Province has the largest number of Neolithic settlement sites.

**Fig 1:**
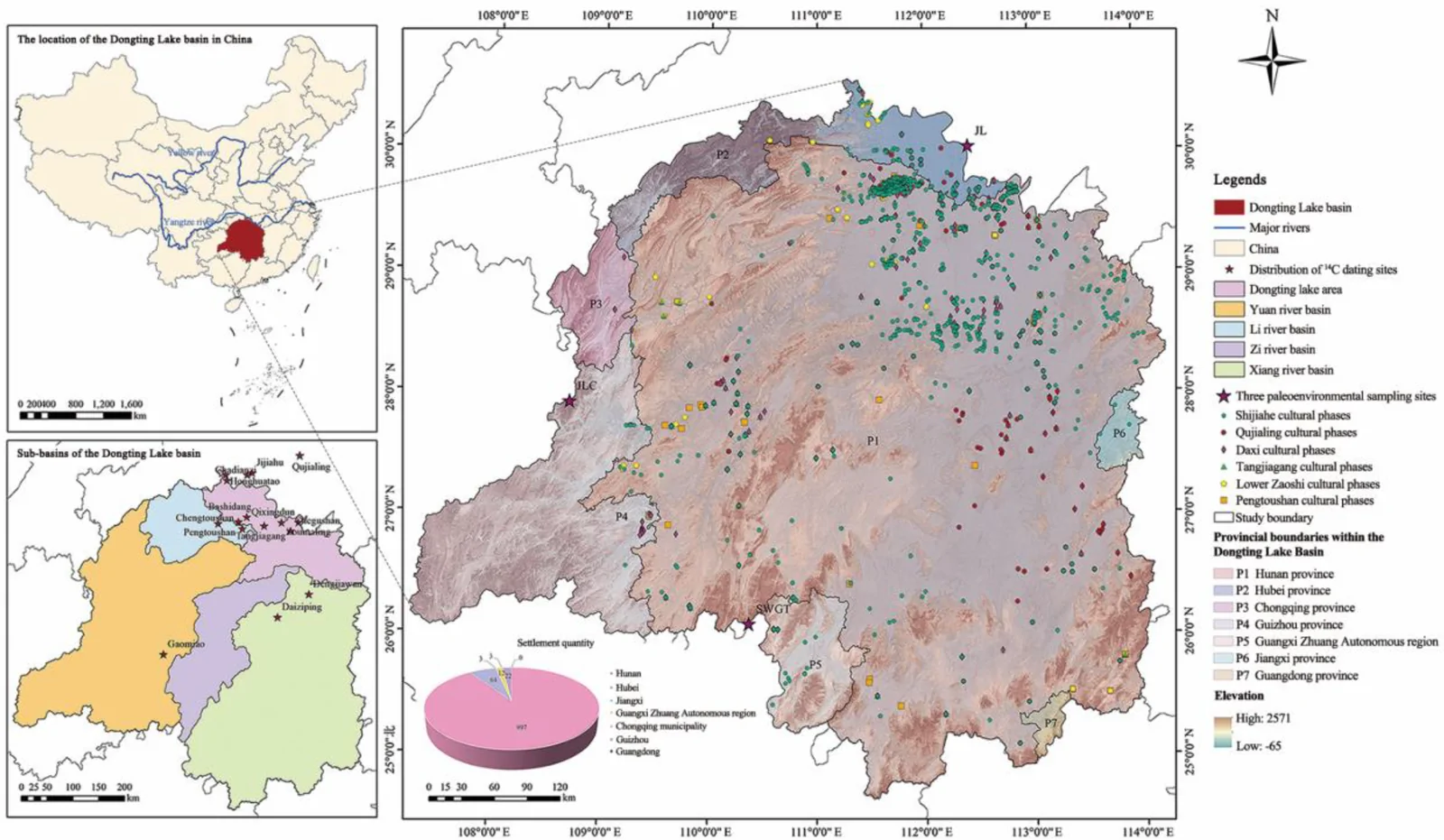
Location of the Dongting Lake Basin. Caption credit: (a) Location of the Dongting Lake Basin in China; (b) Sub-basins of the Dongting Lake Basin and distribution of ^14^C-dated settlement sites; (c) Provincial boundaries within the Dongting Lake Basin and distribution of Neolithic settlement sites by cultural phase (The three paleoenvironmental sampling sites are SWGT, Shiwangutian peatland, JL, Jiangling section; and JLC, Jiulongchi wetland). Note: All base maps of Dongting Lake Basin presented in this article are sourced from the USGS National Map Viewer (http://earthobservatory.nasa.gov/).

### Establishment of a cultural chronology and spatial framework

The Dongting Lake Basin is located in the middle reaches of the Yangtze River. By analyzing the types of specimens recovered from the site and incorporating ¹⁴C-dating data, a cultural chronology has been constructed (Table 1). The Neolithic age in Dongting Lake Basin comprises six cultural phases. The academic community currently identifies the Pengtoushan culture as the earliest Neolithic culture in the middle Yangtze River region, represented by sites such as Pengtoushan and Bashidang, which date to approximately 9.0–7.8 ka B.P. The Lower Zaoshi culture subsequently developed from the Pengtoushan culture, with key sites including Lower Zaoshi and Hujiawuchang, dating to around 7.8–6.8 ka B.P. The Tangjiagang culture represents a transitional phase between the Lower Zaoshi and Daxi cultures. Representative sites such as Chengtoushan and Tangjiagang date to approximately 6.8–6.3 ka B.P. The Daxi culture marked a period of expanded influence and social advancement, with representative sites including Guanmiaoshan, Honghuatao, Chengtoushan, and Gaomiao, dating from about 6.3 to 5.2 ka B.P. The Qujialing culture represents a regional peak, characterized by clear settlement hierarchies. Key sites include Dengjiawan, Zoumaling, Chengtoushan, Qujialing, and Qixingdun, with absolute dates ranging from approximately 5.2 to 4.6 ka B.P. The Shijiahe period signifies both the culmination and the conclusion of Neolithic cultural development in the Middle Yangtze region. During this phase, social structures grew increasingly complex. Representative sites such as Jijiahu, Daiziping, Chadianzi, and Chegushan date to approximately 4.6–4 ka B.P.

**Table 1:** ^14^C dating data from Neolithic settlement sites in the middle Yangtze River region.

Specimen type	Excavation unit	Settlement sites	Archaeological cultural phases	Calibrated age (yr BP, 95%)	Data source
Pottery shards	OxA1280	Pengtoushan	Pengtoushan	9100 ± 200, 8200 ± 200	[22]
Wood, bamboo charcoal	T14②、T14⑥	Pengtoushan	Pengtoushan	7945 ± 170	[23]
Pottery shards	T9⑤	Bashidang	Pengtoushan	7540 ~ 7100	[24]
Pottery shards	T43⑤	Lower Zaoshi	Lower Zaoshi	6920 ± 200	[25]
Wood charcoal	T102⑦	Hujiawuchang	Lower Zaoshi	7190 ± 140	[26]
Wood charcoal	T4④	Hujiawuchang	Lower Zaoshi	7210 ± 110	
Wood charcoal	T102⑤	Hujiawuchang	Lower Zaoshi	6960 ± 100	
Soil	T1030	Chengtoushan	Tangjiagang	6629 ± 896	[27]
Wood charcoal	AT1808⑦	Tangjiagang	Tangjiagang	6355 ± 180	[28]
Wood charcoal	T1④	Guanmiaoshan	Daxi	5605 ± 130	[29]
Wood charcoal	T76⑧	Guanmiaoshan	Daxi	6630 ± 80	
Wood charcoal and sludge	T110⑤	Honghuatao	Daxi	6305 ± 115	[30]
Wood	T6404	Chengtoushan	Daxi	5360 ± 30	[27]
Charcoal particles	T1115⑥	Gaomiao	Daxi	5420 ± 80	[31]
Wood charcoal	T21④	Dengjiawan	Qujialing	5190 ± 80	[32]
Coffin	M26	Zoumaling	Qujialing	4950 ± 80	[33]
Carbon sludge	Group 2 of city wall rammed earth	Chengtoushan	Qujialing	4800 ± 20	[34]
Wood charcoal	T5⑤	Qujialing	Qujialing	5100 ± 160	[35]
Wood charcoal	Late Qujialing phase I cultural layer	Qujialing	Qujialing	4145 ± 100	
Wood charcoal	Late Qujialing phase II cultural layer	Qujialing	Qujialing	4195 ± 160	[36]
Wood charcoal	TS58E06Q2I⑤	Qixingdun	Qujialing	4550 ± 30	[37]
Beef bones	T1F2 mid-level	Jijiahu	Shijiahe	4500 ± 260	[38]
Carbon sludge	T1 third level M3	Daiziping	Shijiahe	3900 ± 85	[39]
Wood charcoal	T1H21	Chadianzi	Shijiahe	3960 ± 140	[40]
Charcoal dust	2016HCTS02W04④	Chegushan	Shijiahe	3880 ± 25	[41]

There are 51 settlement sites in the Pengtoushan cultural phases, 91 settlement sites in the Lower Zaoshi cultural phases, 56 settlement sites in the Tangjiagang cultural phases, 259 settlement sites in the Daxi cultural phases, 277 settlement sites in the Qujialing cultural phases, and 821 settlement sites in the Shijiahe cultural phases.

### Data sources and processing

The data types utilized in this study are primarily classified into two categories: (1) Map data including base maps of China, administrative boundaries, Digital Elevation Models (DEM), water system, and reconstructions of Dongting Lake’s extent across various cultural phases. (2) Spatial attribute data for settlement sites is primarily sourced from the *Atlas of Cultural Heritage Sites in China: Hunan Province Volume* [42]. OSGeo China Historical Map Data Network, and key immovable cultural heritage sites officially designated by the National Cultural Heritage Administration of China. The scale and attribute data for the cultural heritage sites in Linli county and Miluo city are primarily obtained from excavation reports and academic monographs pertinent to settlement sites from various cultural phases within the Dongting Lake Basin (Table 2).

**Table 2:** Data sources and processing.

Data type	Data name	Data source	Data accuracy	Data processing
**Map data**	Map of China	http://earthobservatory.nasa.gov/	1:1,000,000	——
	Administrative boundary		1:4,000,000	Using the “Clip tool” in ArcGIS 10.5 to extract the study area boundary.
	DEM	http://landsat.visibleearth.nasa.gov/	12.5m dpi	Using the “Clip tool” in ArcGIS 10.5, extract the DEM distribution map of the study area and set the projected coordinate system to WGS_1984_UTM_Zone_49N.
	Water system	http://www.openstreetmap.org	1:2,000,000	Using the Mask Extraction tool in ArcGIS 10.5, a distribution map of the water systems within the study area was extracted. ArcGIS hydrological analysis tools were applied to identify major rivers and tributaries in the Dongting Lake Basin, in order to analyze the distance between settlement clusters and water sources.
	Map of the extent of Dongting Lake at different cultural phases	*Atlas of Historical Vicissitude in Dongting Lake*	1:1,100,000	Performing georeferencing and digitizing in ArcGIS 10.5.
**Settlement site attribute data**	Spatial attribute information data	*Atlas of cultural Heritage Sites in China: Hunan Province Volume* —— Map of Neolithic sites in Hunan province	1:3,500,000	Performing georeferencing and digitizing in ArcGIS 10.5.
		https://osgeo.cn/map/m0302/	1:46,20,000,000	
		http://www.ncha.gov.cn/col/col2266/index.html	—	Spatial coordinate positioning of newly documented Neolithic settlement site points in ArcGIS 10.5.
		Cultural heritage sites in Linli county https://www.changde.gov.cn/lccd/whcd/gjxz/sjww	—	
		Cultural heritage sites in Miluo city http://www.mlwww.net/	—	
	Scale attribute information data	*Archaeology*	—	Establishing a settlement site scale attribute database using ArcGIS 10.5.
		*Hunan Archaeology Journal*	—	
		*Archaeology of Hunan (Volumes I and II)*	—	
		*Jianghan Archaeology*	—	
		*A Study of Prehistoric Settlement Patterns in China*	—	
		*Environmental Archaeology Since the Neolithic age in the Yangtze River Valley, China*	—	
		*The Prehistoric Era of the Middle Yangtze River*	—	

The spatial coordinates of cities, counties, townships and villages where the settlement sites are located were identified by using the Baidu map coordinate picking tool, and the spatial geographic coordinates of the settlement sites were uniformly changed to WGS_1984_UTM_Zone_49N and imported into ArcGIS 10.5.

By integrating expert consultations with a systematic review of archaeological reports, historical archives, and specialized research monographs, we synthesized data pertaining to the cultural phases, distribution areas, and associated remains of individual settlement sites. Subsequently, attribute data—including chronological phases and related characteristics—were compiled and entered into ArcGIS, thereby establishing a high-resolution spatiotemporal database for Neolithic settlements in the Dongting Lake Basin (Fig 2).

**Fig 2:**
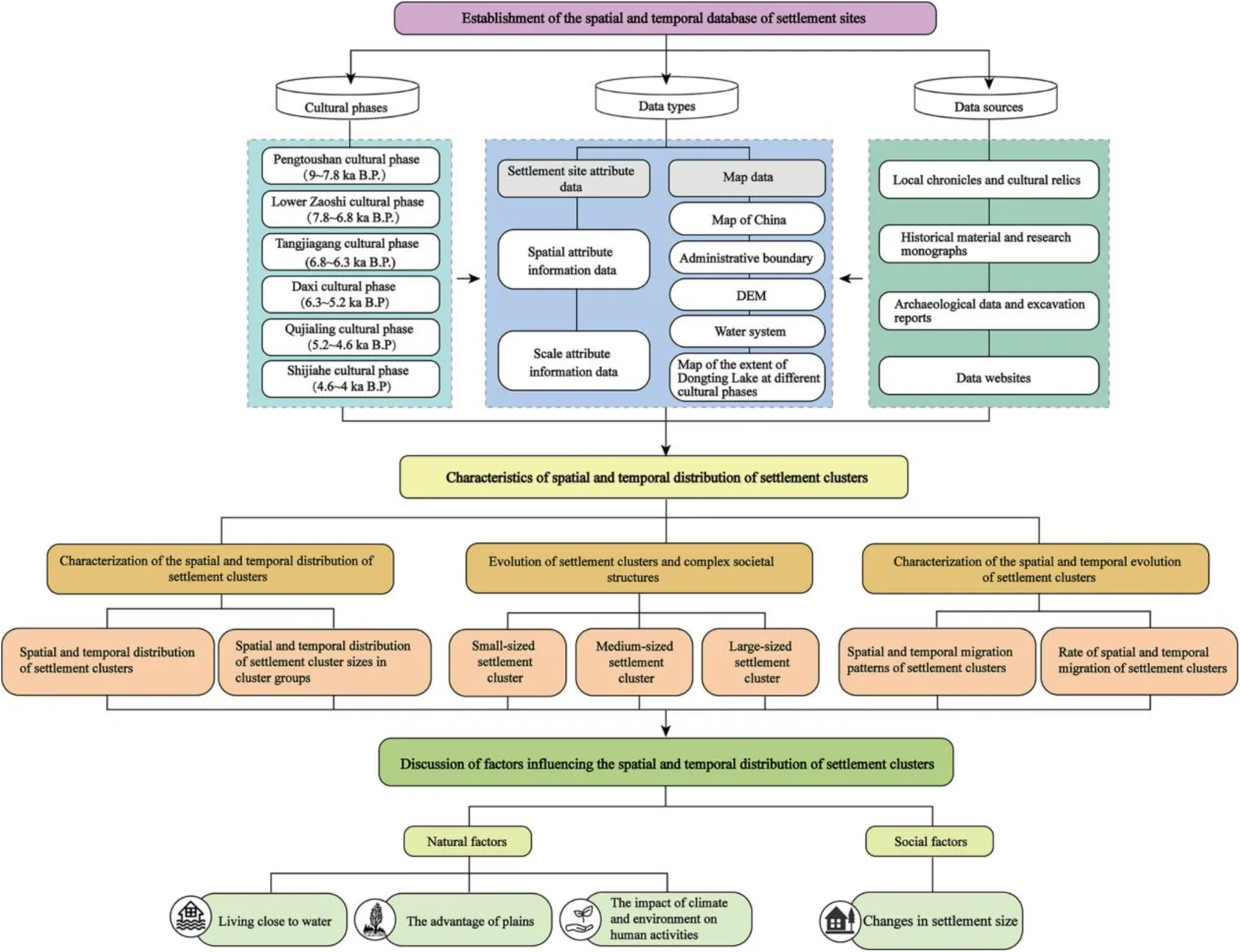
Research framework.

## Research methods

### DBSCAN clustering

The distribution pattern of the settlement sites changed under the effects of different natural and social factors and a settlement cluster was formed when the sites were distributed at a high density. The DBSCAN algorithm was used to explore the distribution pattern of settlement sites located in the Dongting Lake Basin, classify settlement clusters, and analyze their evolution characteristics. Common in cluster analysis are K-means cluster analysis, however, K-means needs to determine the number of clusters before forming clusters, which requires some experience and is characterized by a certain degree of subjectivity [43]. But DBSCAN algorithm is a density-based clustering method for spatial data that accurately categorizes dense areas of sample points into separate cluster. First, the cost distance analysis tool in spatial analysis was used to calculate the cost distance between the settlement sites of different cultural phases and saved in the form of a matrix. Second, by importing this matrix into the DBSCAN program and setting a radius value for the site data of different cultural phases and clustering them, this method reduces the uncertainty caused by subjective judgment to a certain extent.

The core idea is that for each object forming a certain cluster, the number of data objects within a given radius (ε) is higher than a given value (MinPts). The points of the settlement sites for each cultural phase were selected as the base data, and the Euclidean distance between the points was calculated and formed into a matrix. The data were input into the DBSCAN calculation code, and a k-dist plot was used to determine the values of Eps. k-dist indicates the Euclidean distance of the k-th point closest to point P, and the size of k-dist reflects the density of the area where the point is located [44].

The formulae for the neighborhood radius ε and constant value MinPts are as follows:


$$\begin{array}{c} \epsilon =\sum_{k=\text{1}}^{\text{2}\text{0}}{mean}_{i}/k/n \end{array}$$ (1)

$$\begin{array}{c} Minpts=\frac{\text{1}}{n}\sum_{i=\text{1}}^{n}{Count}_{i} \end{array}$$ (2)

where *mean*_*i*_ is the average sum of the nearest distances around *i* settlement site points, *k* is the number of nearest settlement site points adjacent to the core site point, *n* is the total number of settlement sites, and *Count*_*i*_ is the number of points in the neighborhood *i* of any settlement site point [45].

### Spatiotemporal object model

In geoscientific research, modeling complex spatiotemporal dynamics to characterize transitional processes between pre‑ and post‑event states of geographic entities—and to rigorously substantiate and analyze their underlying causal mechanisms—constitutes the most effective paradigm for representing spatiotemporally intricate phenomena [46]. The spatiotemporal evolution characteristics represent changes in the temporal and spatial properties of objects. We established a spatiotemporal data model of the settlement cluster, treated the settlement cluster as a spatial object in each period, and presented the occurrence, development, and extinction of settlement cluster objects, as well as the direction and speed of the changes in the settlement cluster [47], to reveal the state and spatiotemporal evolution process of geographic objects in different periods in the same geographic space.

(1) Settlement center point calculation

Using the cluster centroid factor to mark the state characteristics of the settlement cluster, we could better analyze the speed, direction, and evolution trajectory of the cluster ensemble solution change, and further explore the spatial and temporal evolution patterns of the settlement cluster. The expression for calculating the centroid of cluster (*C*_*x*_, *C*_*y*_) is as follows:


$$\begin{array}{c} \begin{array}{c} C_{x}=\text{(}x1+x2+x3\text{)}/3\\ C_{y}=(y1+y2+y3)/3 \end{array} \end{array}$$ (3)

(2) Calculation of the settlement central point evolution decomposition rate

The distance between the settlement cluster centroids was used as a measure of the decomposition speed of the settlement cluster centroid evolution, and the speed of the change in the settlement cluster distribution was inferred from the change in settlement cluster centroids as representing the changing state of the clusters in the two cultural phases. When considering the center point as a three-dimensional coordinate (*x*_*i*_, *y*_*i*_, *t*_*i*_) with time-informed variables and the velocity of the change in the center of gravity as *v*, we obtain the following formula:


$$\begin{array}{c} v=\frac{\sqrt{(xi+1-xi{)}^{\text{2}}+(yi+1-yi{)}^{\text{2}}}}{ti+1-ti} \end{array}$$ (4)

## Results

### Characterization of the spatial and temporal distributions of settlement cluster

By dividing the settlement clusters of different cultural phases in the Dongting Lake Basin (Table 3), we found that settlement cluster PC in the Pengtoushan cultural phase was mainly distributed in the northwest of Dongting Lake Basin area, in which 22 settlement sites were distributed, with the majority being medium-sized settlements (Fig 3a). 29 sites did not occur in the settlement cluster and were mainly small settlements, no large settlements were identified within these settlement clusters.

**Table 3:** Settlement sizes within settlement clusters across different cultural periods.

Settlement scale types	Settlement cluster in different cultural phases					
	Pengtoushan	Lower Zaoshi	Tangjiagang	Daxi	Qujialing	Shijiahe
	PC	ZC	TC1-TC2	DC1-DC7	QC1-QC7	SC1-SC11
	Number/units	Number/units	Number/units	Number/units	Number/units	Number/units
**Small-sized (0–5000m**^**2**^)	8	5	17	97	97	277
**Medium-sized (5000–10**^**5**^**m**^**2**^)	14	23	12	104	89	257
**Large-sized** **(> 10**^**5**^**m**^**2**^)	0	0	1	3	4	8

**Fig 3:**
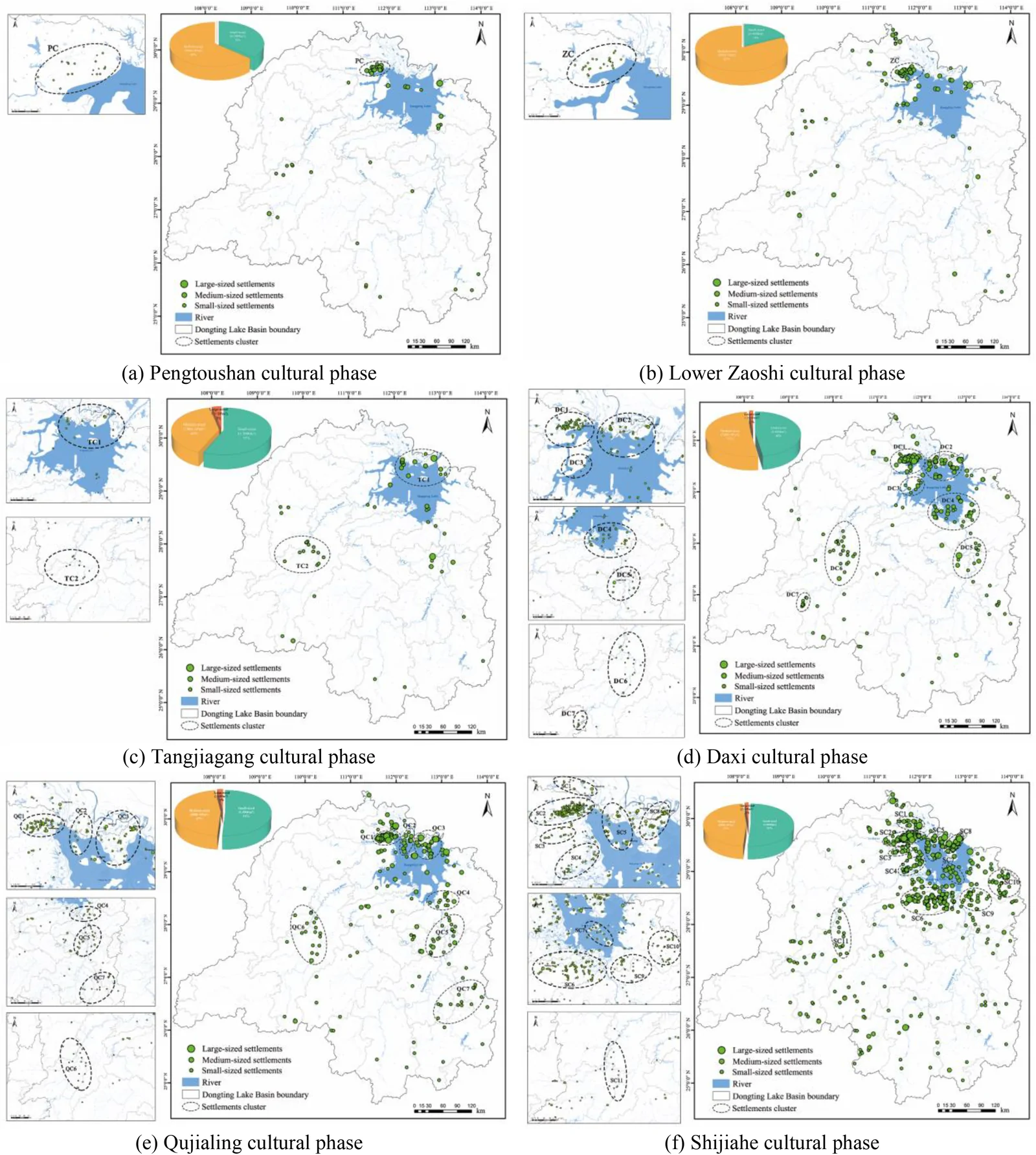
Distribution of settlement clusters in the Dongting Lake Basin during the Neolithic age. (The reconstructed prehistoric boundaries of Dongting Lake are based on the *Atlas of Historical Vicissitude in Dongting Lake* [48]).

The number of settlements distributed around Dongting Lake increased during the Lower Zaoshi cultural phase, with 28 settlement sites occurring in settlement cluster ZC. The PC settlement cluster crossed the Lower Zaoshi cultural phase from its previous period, and the sites occurring in the settlement cluster were also medium-sized, whereas 63 sites did not occur in the settlement cluster during this phase and were mostly small-sized settlements (Fig 3b).

The number of settlements north of Dongting Lake increased during the cultural phase of Tangjiagang. The number of settlements near the Yuanjiang River and its tributaries to the west gradually increased, forming two settlement clusters: 16 settlement sites in TC1 and 14 in TC2 (Fig 3c). A total of 26 settlement sites were distributed independently, Settlement cluster TC1 was dominated by medium-sized settlements and included the large fortified settlement of Qixingdun, whereas settlement cluster TC2 was composed primarily of small settlements.

The number of settlement sites around Dongting Lake increased during the Daxi cultural phase. Settlement clusters DC1, DC2, DC3, and DC4 contained 76, 39, 10, and 35 settlement sites, respectively. Moreover, the DC1, DC2, and DC4 settlement clusters were mainly medium-sized, whereas the DC3 settlement cluster was small-sized. Moreover, fortified settlement sites, such as Youzhougang and Qixingdun, emerged within settlement clusters DC1 and DC2. During this cultural phase, the number of settlement sites gradually increased near the Xiangjiang River and formed settlement cluster DC5, in which 13 settlement sites were distributed, and the settlement size was mainly small. Settlement cluster DC6, which was distributed in the Yuanjiang River and its tributaries, increased in size in comparison with the previous cultural phase and formed a small-sized settlement cluster DC7 to the south, with 22 and 9 settlement sites distributed in DC6 and DC7, respectively; the settlement size was mostly small (Fig 3d).

The number of settlements north of Dongting Lake increased during the Qujialing cultural phase, whereas that in the south decreased compared to the previous cultural phase. Therefore, settlement clusters QC1, QC2, and QC3 formed north of Dongting Lake, the number of medium- and large-sized settlements increased in these clusters. Moreover, the number of large settlements within settlement clusters QC1 and QC3 increased compared with the preceding period. In addition to the two fortified settlement sites already present during the previous period, the Jijiaocheng and Tunzishan sites newly emerged. The number of settlement clusters distributed along the Xiangjiang River and Yuan River increased, QC4, QC5, QC6 and QC7, which were mainly small settlement clusters, formed. The numbers of sites within the settlement cluster from QC1 to QC7 were 66, 12, 44, 12, 24, 14, and 18, respectively (Fig 3e).

The settlement sites distributed around Dongting Lake during the Shijiahe cultural phase multiplied, mainly accumulating around Dongting Lake, forming 11 settlement clusters distributed around the lake. The numbers of sites within the settlement cluster from SC1 to SC11 were 20, 202, 15, 39, 30, 108, 15, 45, 22, 33, and 13, respectively. The number of sites that did not occur in the cluster is 279, and the settlement clusters gathered around Dongting Lake were mainly medium-sized. Moreover, the number of settlement clusters containing large settlements increased, with such settlements occurring in clusters SC1, SC2, SC5, and SC8. In addition to the four large settlement sites identified in the preceding period, four new large fortified settlement sites—Jimingcheng, Sunjiagang, Lubaoshan, and Sanchagang—emerged during this period (Fig 3f).

### Evolution of settlement clusters and complex societal structures

The spatial organization of Neolithic settlements in the Dongting Lake Basin was not static but underwent a gradual transition from dispersed settlement clusters to increasingly centralized and hierarchical configurations. Changes in settlement spatial structure were closely associated with the increasing complexity of regional social organization. During the early stages, or in mountainous areas, settlement clusters were predominantly composed of small settlements of comparable size. As shown in Table 4, the small settlement clusters TC2, DC7, QC7, and SC11 were largely constrained by mountainous terrain. Settlements within these clusters exhibited a relatively high degree of homogeneity in both spatial organization and settlement size, with no clearly dominant central settlements having yet emerged. This spatial configuration, characterized by “small settlements - loose clustering,” suggests that early settlement clusters were associated with a relatively egalitarian form of social organization and an overall low level of social complexity.

**Table 4:** Spatiotemporal distribution and structure of settlement clusters across cultural phase.

Cultural phase	Settlement cluster characteristics	Settlement cluster structure	Settlement cluster distribution
Pengtoushan	Small-sized settlement clusters	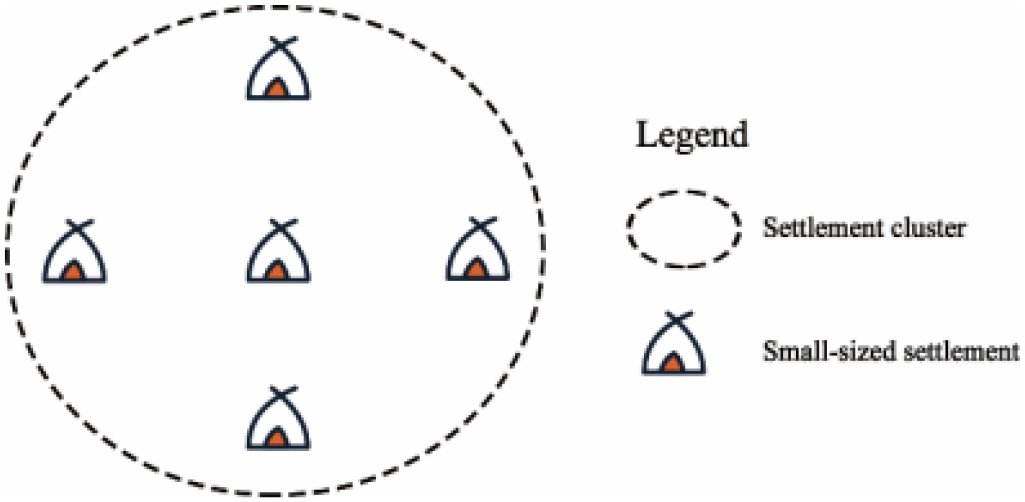	/
Lower Zaoshi			/
Tangjiagang			TC2
Daxi			DC7
Qujialing			QC7
Shijiahe			SC11
Pengtoushan	Medium-sized settlement cluster	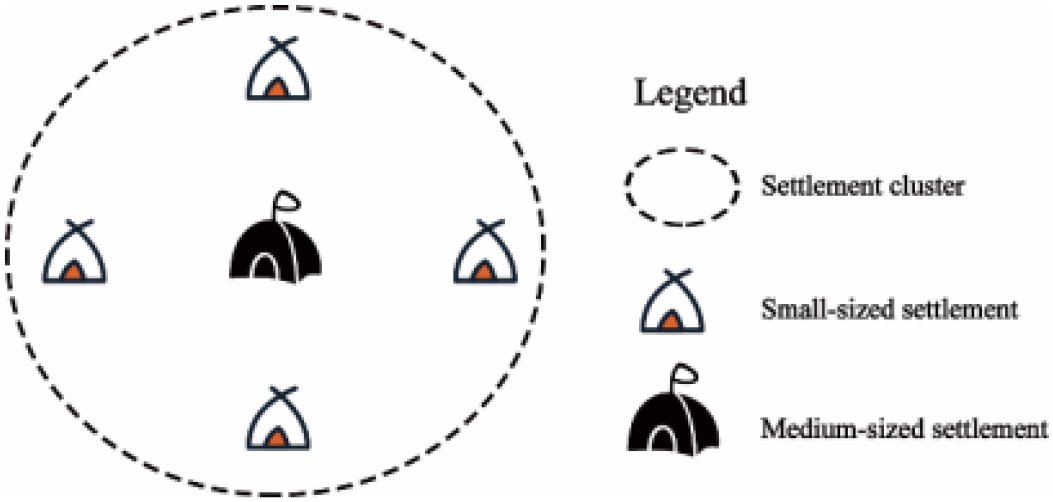	PC
Lower Zaoshi			ZC
Tangjiagang			/
Daxi			DC3, DC4, DC6
Qujialing			QC2, QC4, QC5, QC6
Shijiahe			SC3, SC4, SC7, SC9, SC10
Pengtoushan	Large-sized settlemen cluster	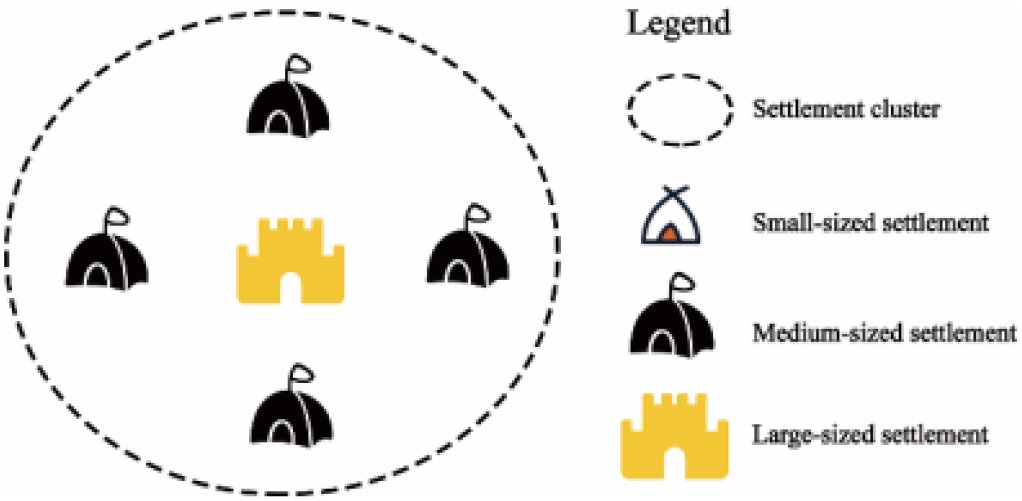	/
Lower Zaoshi			/
Tangjiagang			TC1
Daxi			DC1, DC2, DC5
Qujialing			QC1, QC3
Shijiahe			SC1, SC2, SC5, SC6, SC8

With population growth, the development of agricultural production, and the intensification of social interactions among settlements, the number of settlement clusters increased, while differences in settlement size began to emerge within individual clusters, leading to the gradual differentiation of their formerly homogeneous spatial structures. From the Daxi to the Shijiahe cultural periods, the number of medium-sized settlement clusters increased progressively. These clusters were concentrated primarily in the Dongting Lake area and the Xiang River Basin, where relatively flat terrain and abundant water resources provided favorable conditions for settlement development. Some settlements gained advantages in terms of settlement size, population concentration, and resource organization, and gradually developed into medium-sized settlements, as exemplified by settlement clusters PC, ZC, DC3, DC4, DC6, QC2, QC4, QC5, QC6, SC3, SC4, SC7, SC9, and SC10. Relationships among settlements gradually shifted from relatively horizontal and parallel interactions toward network structures characterized by increasing hierarchical differentiation. Medium-sized settlements may have assumed more important functions in resource allocation, production organization, exchange, and communal activities, thereby strengthening the spatial centripetal tendency within settlement clusters. This stage can therefore be regarded as a transitional phase in the Dongting Lake Basin, during which settlement society evolved from relatively egalitarian local-level organization toward an incipient hierarchical social structure.

During the middle-to-late Neolithic period, particularly the Daxi, Qujialing, and Shijiahe cultural periods, the numbers of medium- and large-sized settlements within settlement clusters increased, accompanied by further differentiation in settlement size and a strengthening of spatial hierarchy. Large central settlements, substantially larger than surrounding settlements and characterized by fortified features, also began to emerge, as exemplified by settlement clusters TC1, DC1, DC2, DC5, QC1, QC3, SC1, SC2, SC5, SC6, and SC8. These developments gradually gave rise to a multi-level spatial hierarchy consisting of “large central settlements–medium-sized secondary settlements–small local settlements” [49]. Differences in settlement size no longer merely reflected variations in population size and settlement area, but may also have indicated an increasingly uneven distribution of access to resources, social power, public facilities, production organization, and regional exchange networks. In particular, the emergence of large central settlements suggests an increasing concentration of population and resources at a limited number of core nodes, accompanied by increasingly pronounced functional differentiation and hierarchical relationships among settlements.

### Characterization of the spatial and temporal evolution of settlement clusters

The center of the settlement cluster shifted slightly southward from the Pengtoushan to Lower Zaoshi cultural phases (Fig 4a), and the number of sites in settlement clusters PC and ZC during these two phases differed little, with an increase of only seven sites. Additionally, the evolution from the Pengtoushan to the Lower Zaoshi cultural phases was 2200 a, with a central point coalescence rate of 0.90 m/a (Table 5 and S1 Table).

**Table 5:** Coalescence rates of Neolithic settlement cluster centers.

Number	①	②	③	④	⑤	⑥	⑦	⑧	⑨	⑩
**rate (m/a)**	0.90	56.69	165.92	6.84	54.55	53.97	63.07	124.48	4.18	85.13
**Number**	⑪	⑫	⑬	⑭	⑮	⑯	⑰	⑱	⑲	⑳
**rate (m/a)**	2.82	36.80	37.88	14.47	17.44	20.66	57.13	45.79	77.71	5.01
**Number**	㉑	㉒	㉓	㉔	㉕	㉖	㉗	㉘	㉙	㉚
**rate (m/a)**	78.41	4.38	23.82	30.08	57.48	42.90	52.71	11.89	41.62	65.81
**Number**	㉛	㉜	㉝	㉞	㉟	㊱	㊲			
**rate (m/a)**	54.73	54.38	7.87	66.22	65.90	66.34	8.82			

**Fig 4:**
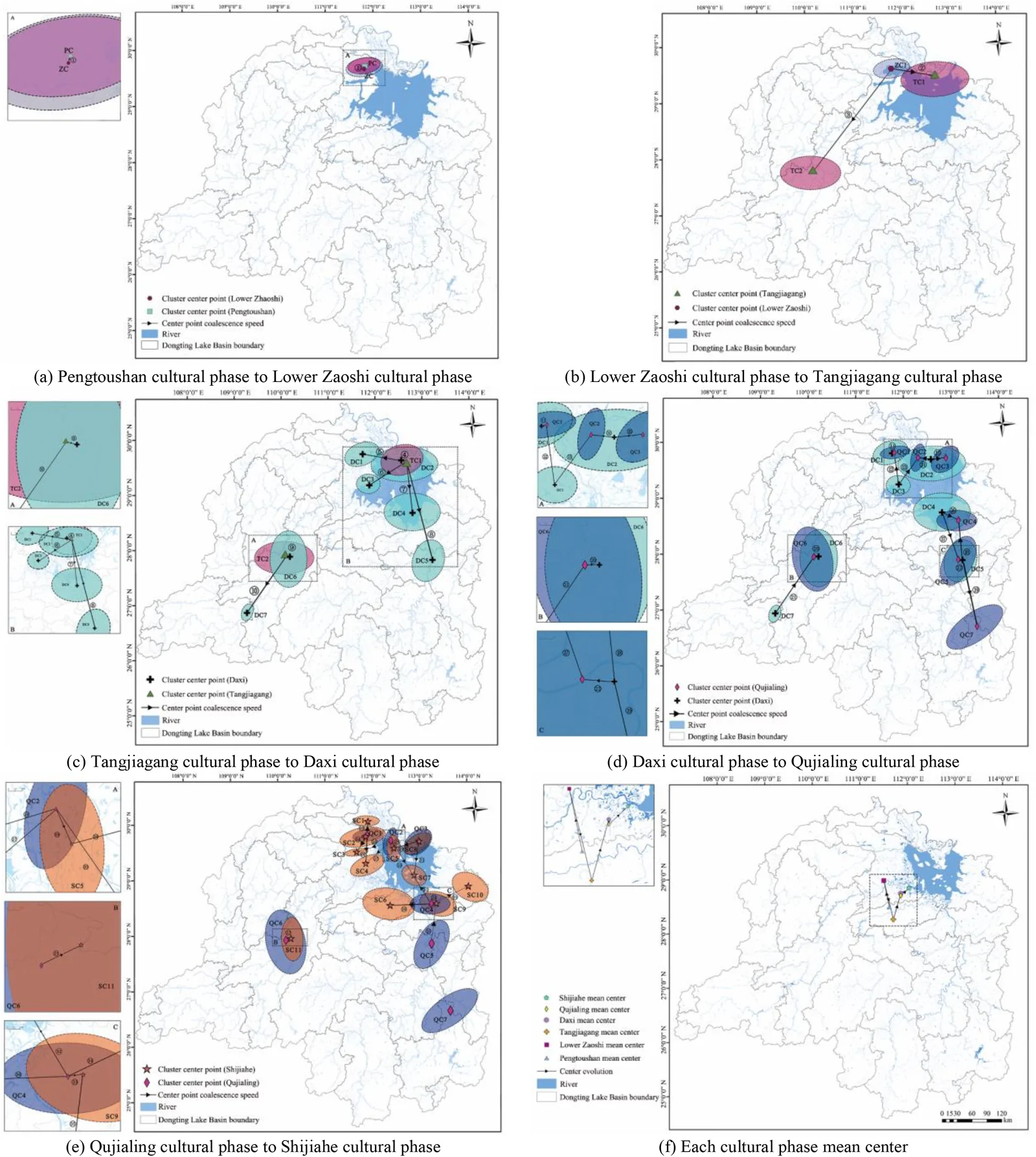
Spatial differentiation and migration of Neolithic settlement cluster centers.

The Tangjiagang cultural phase did not inherit the PC and ZC clusters from the two previous cultural phases but expanded eastward to the vicinity of Dongting Lake (Fig 4b). The evolution from the Lower Zaoshi to the Tangjiagang cultural phase was *t*_*i+1*_ *− t*_i_ = 1500 a. The center point of settlement cluster ZC expanded southwest of TC1 during the Tangjiagang cultural phase, and the center point of settlement cluster ZC concerning the center point of TC1 had a coalescence rate of 56.69 m/a, while the rate of merger of the center point of settlement cluster ZC to that of TC2 was 165.92 m/a, which indicates that the expansion rate of the center point to the southwest was faster than it was to the east. The overall migration rate of the settlement center in the Tangjiagang cultural phase was significantly faster than the previous period, but the monocentric colony cluster form predominated.

The settlement cluster located around Dongting Lake increased in Daxi culture (Fig 4c), and it had a polycentric competitive settlement cluster form. The evolution from the Tangjiagang to the Daxi cultural phase was ti + 1 − ti = 1600 a. The coalescence speed of settlement cluster TC1 and the center point of DC2 was 6.84 m/a, while the central coalescence rate of settlement cluster TC1 toward DC1, DC3, DC4, and DC5 were 54.55 m/a, 53.97 m/a, 63.07 m/a, and 124.48 m/a, respectively, and the central dissolution velocities increased sequentially.

It showed a multi-centered competitive settlement cluster pattern in the Qujialing cultural phase (Fig 4d). The evolution from the Daxi to Qujialing culture phases were *t*_*i+1*_ *− t*_*i*_ = 1700 a. the coalescence speed of the center points of the settlement clusters DC1 and QC1, at 2.82 m/a; the coalescence rate of the settlement cluster DC2 center to the QC2 and QC3 centers were 14.47 m/a and 17.44 m/a, respectively, while the coalescence speeds of the center points of the settlement clusters DC4 and DC5 expanding to the centers of QC4, QC5, and QC7 were 20.66 m/a, 4.38 m/a, 57.13 m/a, 45.79 m/a, and 77.71 m/a, respectively. The expansions of the center of the settlement clusters DC6 and DC7 to QC6 were 5.01 m/a and 78.41 m/a, respectively, thus indicating that the expansion of settlement clusters to the north was significantly lower than the expansion to the south.

The settlement clusters of the Shijiahe cultural phase exhibited a polycentric and competitive pattern. The number of sites within the settlement cluster was nearly three times that in the previous period, while the settlement area in this period was smaller than that in the previous period, with a clear tendency for the settlement cluster to cluster around Dongting Lake (Fig 4e). The evolution from the Qujialing to the Shijiahe cultural phase was *t*_*i+1*_ *− t*_*i*_ = 1200 a. In the two periods, the centroid coalescence rate of the settlement cluster QC1 and SC1, SC2, SC3, and SC4 were 23.82 m/a, 30.08 m/a, and 42.90 m/a, respectively, and the location of the centroids of the settlement clusters QC1 and SC2 were the same. The central coalescence rate of the settlement cluster QC2 with SC3, SC4, SC5, and SC7 were 57.48 m/a, 52.71 m/a, 11.89 m/a, and 65.81 m/a, respectively, which was faster overall than the coalescence toward the northwest of Dongting Lake, and the central coalescence rate of the settlement cluster QC3 with SC5 and SC7 were 41.62 m/a and 54.73 m/a, respectively. The coalescence rate of the settlement clusters QC4 and QC5 to the centers of SC6, SC7, SC9, and SC10 was 260.71 m/a overall, and the coalescence rate of the settlement cluster QC6 to the center of SC11 was 8.82 m/a.

The settlement mean centers across cultural phases were primarily located south of Dongting Lake. They initially migrated from the southern part of Dongting Lake toward the northwest, and subsequently shifted from the south toward the northeast. The overall migration pattern of the settlement centers formed a “V-shape” (Fig 4f). The settlement cluster expanded faster to the south of Dongting Lake, the settlement cluster center located around Dongting Lake was the second fastest, and the settlement cluster located in the western part of Dongting Lake was the slowest.

## Discussion

Due to the prolonged adaptation of early humans to the natural environment, low productivity levels, and limited means of subsistence, communal living patterns gradually emerged. As populations increased, small settlement clusters slowly evolved into colonies. These colonies further expanded into clusters of settlements under the influence of regional environmental conditions and social production factors. Therefore, based on an analysis of the spatiotemporal evolution characteristics of settlements, it is essential to further examine the development of Neolithic settlements in the Dongting Lake Basin from the perspectives of both nature and social factors.

### Natural influence factors on the spatial and temporal evolution of settlement clusters

#### Living close to water.

As can be seen from Fig 5 and S2 Table, the number of settlements within the cluster of settlements in different cultural phases was distributed in the range of 0-5000m from the water in the largest number, which indicates that people in the Neolithic age tended to live within one hour’s walking distance from the water source. This was conducive to the use of water for daily life, fishing and hunting, and irrigation of farmland, while the river also carried the role of social and cultural exchanges [50]. However, from the Daxi cultural phase to the Shijiahe cultural phase, the ratio of the number of settlements within the settlement clusters distributed within the range of 0–5000 m gradually decreased, and some of the settlement clusters gradually migrated to areas farther away from water, thereby indicating that the population and number of settlements increased, the area of arable land expanded, and the existing land did not support the rapid growth of population and arable land, whereby some settlements moved further away from large sources of water.

**Fig 5:**
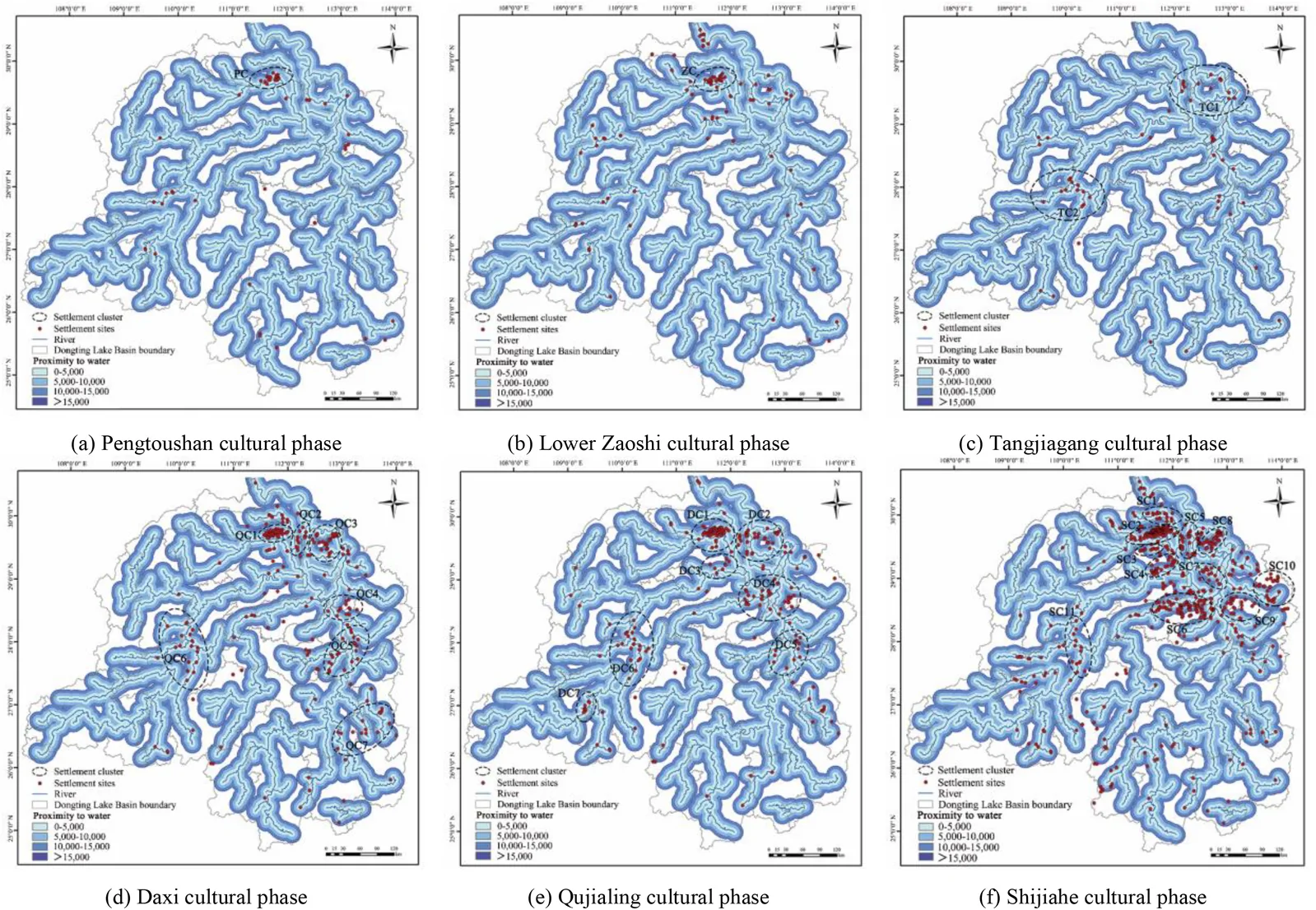
Spatial distribution of settlement clusters relative to water sources across different cultural phase.

The settlement clusters are also characterized by distribution along rivers and lakes. Fig 6 shows that the largest number of settlement clusters are distributed along the periphery of Dongting Lake and the river, the clusters distributed in the Dongting Lake area began to appear in the Tangjiagang cultural phase and increased in the Daxi cultural phase, whereas the settlement cluster distributed in the Li river basin appeared as early as the Pengtoushan cultural phase. Although the number of settlement clusters was smaller than that in the Dongting Lake area, the overall number of settlements within the settlement clusters was higher than that in the Dongting Lake area, thus suggesting that clusters had already formed in the Li river basin during the early Neolithic age [51]. This is attributable to the dense fluvial networks and abundant water resources characterizing both the Dongting Lake region and the Li river basin, coupled with their predominantly river-alluvial plain topography and highly fertile soils. These conditions not only supported productive agricultural practices and facilitated inland waterway transport but also fostered extensive cultural exchange, thereby establishing these two areas as pivotal hubs of Neolithic cultural interaction and development within the broader Dongting Lake Basin.

**Fig 6:**
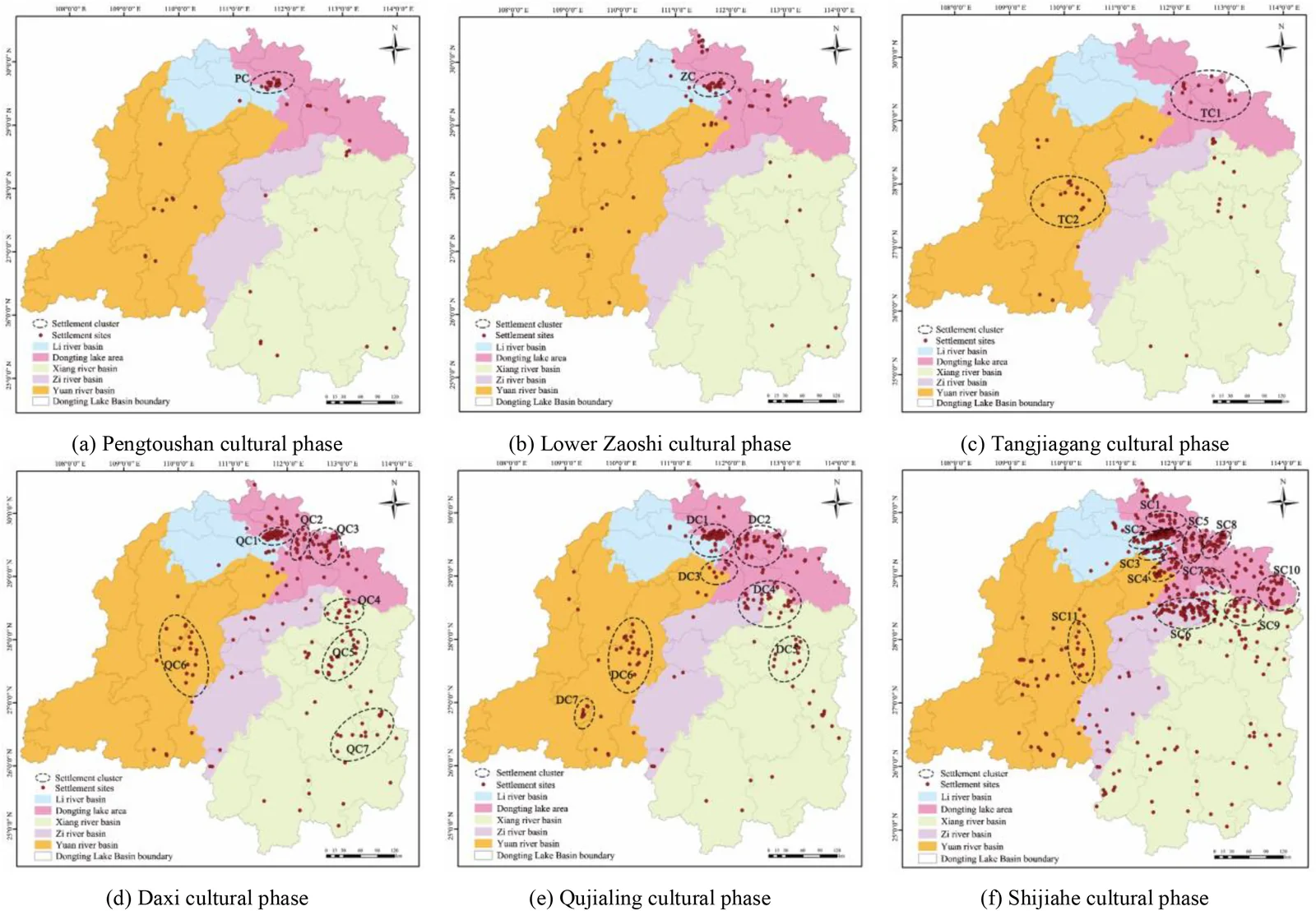
Distribution characteristics of settlement clusters across sub-basins in different cultural phases.

#### The advantage of the plains.

According to the relevant elevation division basis, 0-100m is plain, 101-200m is tableland, 201-500m is hill, > 500m is mountain [52]. Settlement clusters tended to be distributed throughout the plains. As Fig 7 and S2 Table reveals, settlements in the Dongting Lake Basin in each cultural phase were mainly distributed in the plains at an elevation of 0–100m, and the number of settlements here doubled from the Daxi cultural phase to the Shijiahe cultural phase. However, from the Tangjiagang to the Shijiahe cultural phases, there was a marked increase in settlements across tablelands, hills, and mountainous zones. This trend suggests that settlement capacity in the plains had approached saturation by the mid-to-late Neolithic. Intensive rice agriculture practiced on the plains, supported by stable subsistence resources, sustained continuous demographic growth, which in turn drove the rapid spatial expansion and quantitative proliferation of settlement clusters [53].The rapid expansion and shrinkage of land resources led to the gradual migration of many settlements to hilly and mountainous areas, and the separation of some smaller settlements from the larger ones to obtain more resource conditions [54].

**Fig 7:**
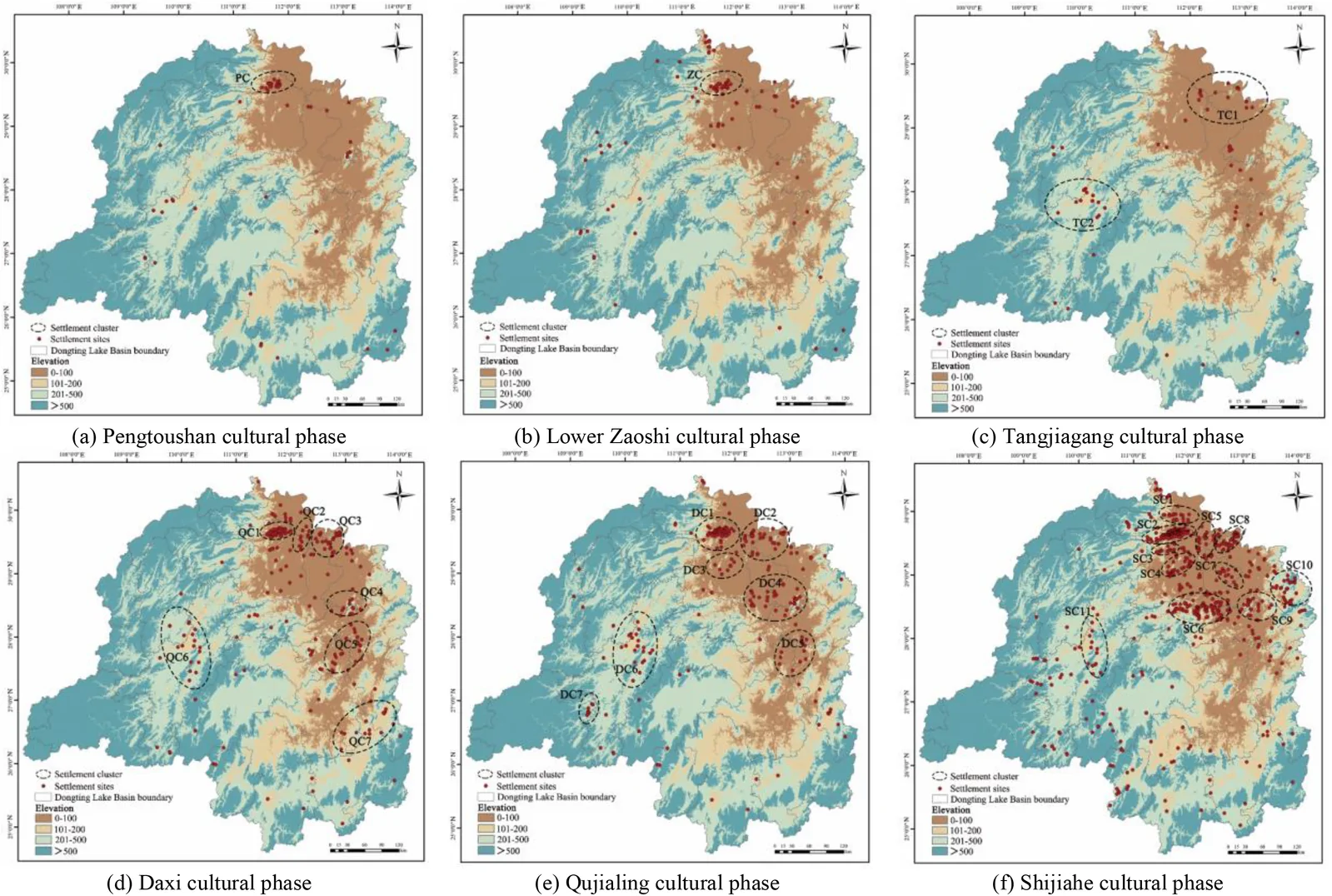
Elevation distribution patterns of settlement clusters across different cultural phase.

Early settlement cluster formation was influenced by topography and environment [55].The settlement clusters were distributed in the northwest of Dongting Lake, which belongs to the pre-mountain area of the Wuling Mountains, with flat terrain and the river and its tributaries providing abundant water for settlements, a large space for the settlements to spread out.

#### The impact of climate and environment on human activities.

Zhao et al [56] reconstructed the Human Intensity Indicators (HII) for the SWGT peat located in the southern part of the Dongting Lake Basin, the northern Hubei JL profile, and the JLC wetland distributed in the western part. From Figs 8a–8c, it can be seen that the intensity of human activities in the middle Holocene was high, especially in the sporulation records of Jiangling area in the northern part of Dongting Lake in the Pengtoushan cultural phase and the Lower Zaoshi cultural phase, which was the highest intensity of human activities. This is due to the fact that the area is located in the northwestern part of Dongting Lake, along the Yangtze River, and has a relatively low topography, abundant water sources, and suitable climate.

**Fig 8:**
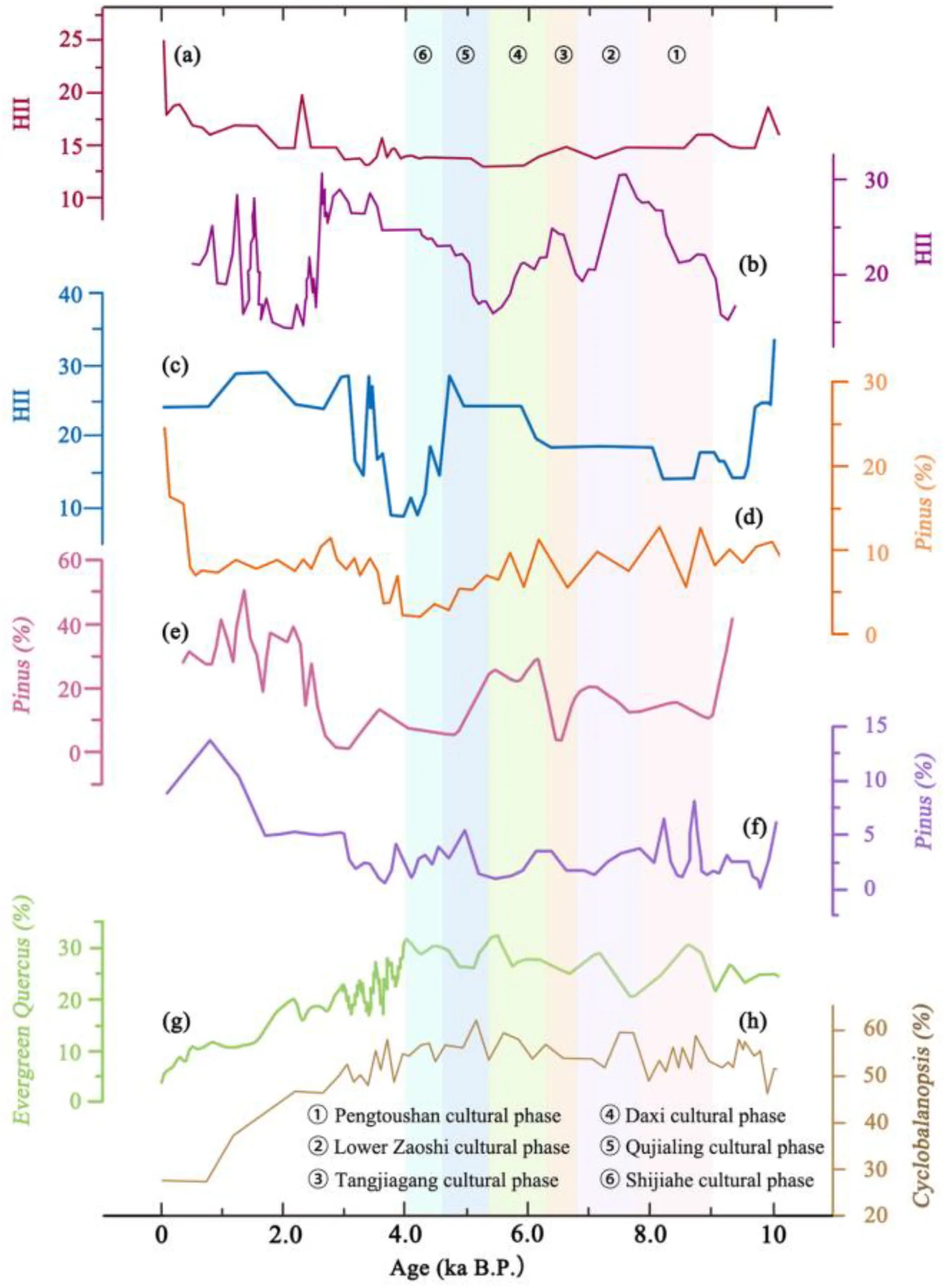
Comparison of pollen records and HII in the Dongting Lake Basin. (a) Temporal variations in HII at the SWGT peatland; (b) temporal variations in HII in the JL profile; (c) temporal variations in HII at the JLC wetland; (d) percentage of *Pinus* pollen in the SWGT peat record; (e) percentage of *Pinus* pollen in the JL profile; (f) percentage of *Pinus* pollen in the JLC wetland record; (g) percentage of evergreen *Quercus* pollen in the SWGT peat record; and (h) percentage of *Cyclobalanopsi*s pollen in the JLC wetland record.

However, periods of climatic deterioration had a direct impact on human activity and settlement stability. For example, during the middle to late Daxi cultural phase, cooling trends and environmental degradation reduced resource availability, which is reflected in the decline of HII. This climatic stress likely triggered migration from less favorable areas, promoted the consolidation of settlements in areas with better water and soil conditions.

Qu et al [57] recorded the sporulation content of *Pinus* in Hunan’s SWGT showing a high percentage of *Pinus* pollen content in the mid- Holocene, suggesting that extensive human logging of broadleaf forests at this time led to the expansion of secondary *Pinus* Forests (Figs 8d-8f). Meanwhile, increased grass content indicates that human activities began to engage in agriculture and settlement in low-altitude areas [58]. Additionally, evergreen *Quercus*, percentage content is higher, indicating that the climate is warm and humid at the mid-Holocene (9.0-4.0 ka B.P.) [59], suitable for the growth of subtropical evergreen broad-leaved forests (Fig 8g). During the 6.0-5.0 ka B.P., the proportion of *Cyclobalanopsi*s increased significantly, reaching a minor peak (Fig 8h), indicating the climate is the warmest and wettest, marking the emergence of the Holocene Optimum period. However, during the 5.0-4.0 ka B.P., the proportion of *Cyclobalanopsi*s declined, and the climate environment underwent fluctuation.

The results of the JLC human activity spore records from the western Dongting Lake Basin indicate that human activity intensity was higher during the period from approximately 6.0 to 4.5 ka B.P., suggesting an increase in population, as well as a growth in the number and clustering of settlement clusters in the region. In addition, the interplay between favorable climatic periods and natural hazards is also reflected in the intensification of agricultural activities. During the Qujialing and Shijiahe cultures (5.5-4.0 ka B.P.), despite the occurrence of localized flooding, the warmest and wettest climate facilitated the expansion of agriculture, population growth, and the development of high-density settlement clusters [60]. This suggests that while natural disasters could disrupt communities, the overall Holocene climate provided sufficient resilience and resources to enable recovery and continued settlement expansion.

### Social influence factors on the spatial and temporal evolution of settlement cluster

#### Changes in settlement size.

Due to the generally warm and humid climate in the middle Holocene, the number of settlements continued to increase over time, and their size increased. By comparing the number of settlement sites in each cultural phase and the time it took, we can get the growth rate of settlements in different cultural phases. The cultural phase from Pengtoushan to Lower Zaoshi lasted about 2200 a, with a total of 142 settlement sites, and on average a new settlement was born about every 15 a. The Lower Zaoshi to Tangjiagang cultural phase lasted 1500 a, with a total of 147 settlement sites, averaging the birth of a new settlement about every 10 a, with a growth rate 1.5 times faster than that of the previous period. During the Tangjiagang and Daxi cultural phases, which lasted about 1600 a, the total number of settlement sites was 315, and a new settlement was born on average about every 5 a, with a growth rate twice as fast as that of the previous period. During the Daxi and Qujialing cultural phases, which lasted about 1700 a, the total number of settlement sites was 536, with an average of about one new settlement being born every 3 a, a growth rate of 1.6 times that of the previous period, and during the Qujialing and Shijiahe cultural phases, which lasted about 1200 a, the number of settlement sites totaled 1,098, with an average of about one new settlement being born every 1 a, a growth rate of three times that of the previous period. This shows that over time, the number of settlements grew and the rate of new settlement generation accelerated, especially during the Shijiahe cultural phase, when the number of settlements and the generation of new settlements increased at a multiplier rate.

Driven by rapid settlement expansion and sustained population growth, decrease in average survival space, escalating survival pressures have emerged as a direct consequence of increasing settlement scale (Fig 9). Based on the number and area of settlement sites, the average living space of settlements in each cultural phase (total area of settlement sites/number of settlement sites) was calculated, and the average area of settlement sites was the highest in the Shijiahe cultural phase, which was nearly 16 times higher than that in the Pengtoushan cultural phase. Taking the total area of the settlements in each cultural phase as the measuring scale for calculating the total population, setting the population index of the Pengtoushan cultural phase as 1, the ratio of the change of the total population in each period (total area of the settlements in each period/total area of the settlements in the Pengtoushan cultural phase) has the value of 1:2.11:1.62:6.36:7.10:15.85, which indicates that the total population of the various cultural phases expanded continuously with the growth of time. Setting the survival space area index of the Pengtoushan cultural phase as 1, the survival area change index of each cultural phase (average survival space area of each settlement site in each cultural phase/average survival space area of each settlement site in the Pengtoushan cultural phase) is 1:1.17:1.47:1.25:1.30:0.99, which shows that with the increase in the number of settlement sites and population, the average survival space possessed by the Shijiahe cultural phase sites have a rapidly decreasing average subsistence space. The human survival pressure index (ratio of total population change in each period/index of survival area change in each cultural phase) is 1:1.80:1.10:5.08:5.45:15.92, which indicates that the human survival pressure was higher in the Late Neolithic Shijiahe cultural phase, implying that nearly three times as much production was needed to satisfy the survival needs of the people in the Shijiahe period on a similar piece of land needs.

**Fig 9:**
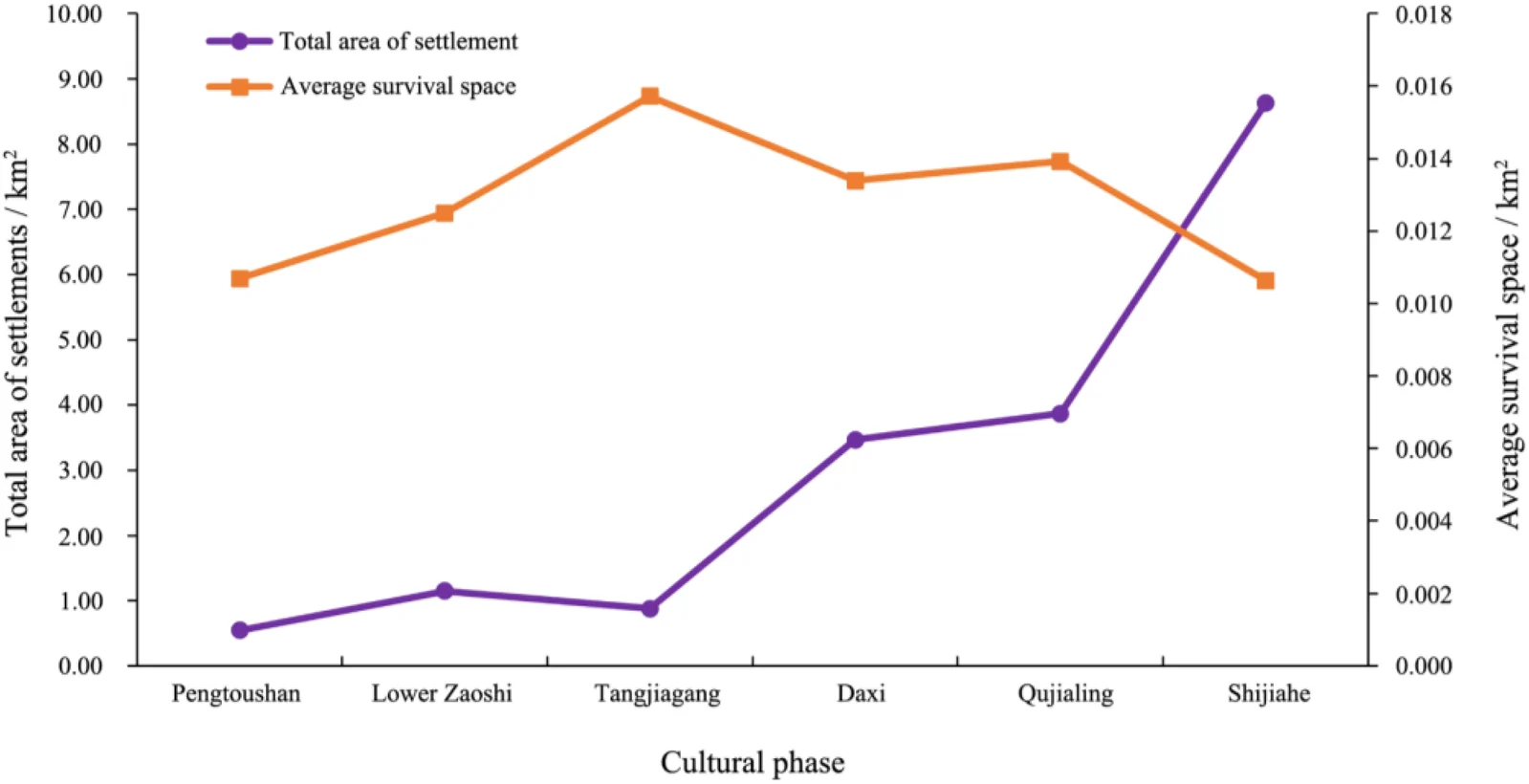
Overall changes in settlement size during the Neolithic age.

### Research limitations and future prospects

This study examines the spatial distribution patterns of Neolithic settlement clusters in the Dongting Lake area by analyzing their spatiotemporal characteristics. It explores the evolutionary trajectories of these clusters across different cultural phases from the perspectives of both natural and social environments. However, the settlement site data analyzed in this study are mainly derived from publicly available archaeological sources for the purpose of data collection and statistical analysis. As archaeological excavations progress, and considering the spatial, methodological, and technical variations in cross-regional excavation practices, future research is expected to improve the accuracy of settlement site information. This will allow for a more comprehensive and systematic understanding of the spatial evolutionary characteristics and influencing mechanisms of settlement clusters.

## Conclusions

Prehistoric settlements provide importance archaeological evidence for understanding the origins and development of Chinese civilization. Investigating their spatiotemporal organization and evolutionary patterns can offer valuable insights into the formation and transformation of prehistoric settlement systems and the development of social complexity. Taking the Dongting Lake Basin in the middle reaches of the Yangtze River as the study area, this study integrated DBSCAN, an object spatiotemporal model combined and ArcGIS-based spatial analysis to reconstruct the spatiotemporal evolution of prehistoric settlement clusters and identify changes in their spatial organization over time. The main findings can be summarized as follows:

The spatial distribution of settlement clusters exhibited a clear temporal shift throughout the Neolithic period. From the Pengtoushan to the Lower Zaoshi cultural phase, settlement clusters were predominantly concentrated in the northwestern part of Dongting Lake Basin. From the Tangjiagang cultural phase, their distribution gradually expanded from the areas surrounding Dongting Lake toward the south. Subsequently, from the Daxi to the Shijiahe cultural phase, the number settlement clusters increase markedly, with an increasingly pronounced concentration around Dongting Lake. Overall, these changes indicate a progressive transformation of the regional settlement system from relatively localized and dispersed distributions toward broader spatial expansion and intensified clustering.

The spatial organization of settlement clusters gradually evolved from a pattern characterized by “small settlements and loose clustering” into a multi-level hierarchy consisting of “large central settlements–medium-sized secondary settlements–small local settlements.” Meanwhile, the spatial center of settlement clusters shifted predominantly southward at a relatively rapid pace, with comparatively slower shifts toward the northwestern part and the peripheral areas of Dongting Lake. Overall, the migration trajectory of the settlement clusters exhibited a distinct V-shaped pattern.

With the development of rice agriculture, settlement clusters tended to be located in areas close to water and on flatter terrain. The Neolithic period broadly coincided with the Holocene Optimum, during which the Dongting Lake Basin was characterized by extensive forest cover, intensified monsoonal activity, and a warm and humid climatic. Evergreen Broad-leaved forests constituted the dominant vegetation, while the northwestern part of the basin showed relatively pronounced evidence of anthropogenic activity. Favorable climatic conditions, together with the development of rice agriculture, facilitated an increase in the number of settlement and the formation of settlement clusters. Concurrently, increasing pressure migration among settlement clusters, thereby contributing to the increasing social complexity and development of prehistoric societies in the middle Yangtze River valley.

## Supporting information

S1 Table Coordinates of settlement cluster centers. This table provides the X and Y coordinates of settlement cluster centers across different cultural phase.
(XLS)

S2 Table Neolithic age settlement site data. This table provides the X and Y spatial coordinates, water distances, and elevation data for 1,101 Neolithic settlement sites distributed in the Dongting Lake Basin.
(XLS)
